# Should we embed randomized controlled trials within action research: arguing from a case study of telemonitoring

**DOI:** 10.1186/s12874-016-0175-6

**Published:** 2016-06-08

**Authors:** Karen Day, Timothy W. Kenealy, Nicolette F. Sheridan

**Affiliations:** Health Systems, School of Population Health, Faculty of Medical and Health Science, The University of Auckland, 261 Morrin Road, St Johns, Auckland New Zealand; Departments of Medicine and General Practice & Primary Health Care, Faculty of Medical and Health Science, The University of Auckland, Auckland, New Zealand; School of Nursing, The University of Auckland, Auckland, New Zealand

**Keywords:** Telehealth, RCT, Action research, Multiparadigm inquiry, Telemonitoring

## Abstract

**Background:**

Action research (AR) and randomized controlled trials (RCTs) are usually considered to be theoretically and practically incompatible. However, we argue that their respective strengths and weaknesses can be complementary. We illustrate our argument from a recent study assessing the effect of telemonitoring on health-related quality of life, self-care, hospital use, costs and the experiences of patients, informal carers and health care professionals in two urban hospital services and one remote rural primary care service in New Zealand.

**Methods:**

Data came from authors’ observations and field notes of discussions with three groups: the healthcare providers and healthcare consumers who participated in the research, and a group of 17 researchers and collaborators. The consumers had heart failure (Site A, urban), airways disease (Site B, urban), and diabetes (Site C, rural). The research ran from 2008 (project inception) until 2012 (project close-off). Researchers came from a wide range of disciplines. Both RCT and AR methods were recognised from early in the process but often worked in parallel rather than together. In retrospect, we have mapped our observed research processes to the AR cycle characteristics (creation of communicative space, democracy and participation, iterative learning and improvement, emergence, and accommodation of different ways of knowing).

**Results:**

We describe the context, conduct and outcomes of the telemonitoring trial, framing the overall process in the language of AR. Although not fully articulated at the time, AR processes made the RCT sensitive to important context, e.g. clinical processes. They resulted in substantive changes to the design and conduct of the RCT, and to interpretation and uptake of findings, e.g. a simpler technology procurement process emerged. Creating a communicative space enabled co-design between the researcher group and collaborators from the provider participant group, and a stronger RCT design.

**Conclusions:**

It appears possible to enhance the utility of RCTs by explicitly embedding them in an AR framework to shape stronger RCT design. The AR process and characteristics may enable researchers to evaluate telehealth while enhancing rather than compromising the quality of an RCT, where research results are returned to practice as part of the research process.

**Trial registration:**

Australian New Zealand Clinical Trials Registry, reference ACTRN12610000269033.

## Background

Good clinical decisions require evidence on more than just effectiveness [1]. Clinicians, consumers, and health systems, need evidence about processes, social context, patient engagement, equity, and health literacy; such factors are typically and explicitly eliminated from Randomised Controlled Trial (RCT) designs as sources of bias and confounding. Interventions whose effectiveness may vary with context, such as information and communication technologies (ICT), and many other health services interventions, challenge the ability to reduce bias and even the wisdom of attempting to do so.

Multiparadigm inquiry offers different ways of knowing [2], which in turn, offer opportunities to learn about factors excluded from positivist RCT reports. Rich data aiming at a thick description as described by Geertz [3] that includes nuanced, multi-perspective, qualitative and quantitative data can be collected and used, e.g., to offer explanations for RCT findings and identify unanticipated consequences of interventions [4]. Action Research (AR), in contrast to RCTs, consists of cycles of planning, acting, doing, and adjusting the plan, as well as characteristics such as [1] participation and democracy, [2] communicative space, [3] iterative improvement, [4] emergence, and [5] different ways of knowing [5]. AR naturally falls within critical and interpretivist approaches [6] and aims at change. RCT and AR are therefore well placed for theoretical pluralism because what is weak in one is strong in the other for the purpose of evaluating complex healthcare interventions such as telemonitoring, as outlined in Table 1.

**Table 1:** Strengths and weaknesses of RCTs and Action Research

	RCT	Action Research
Epistemology (positivist, critical, interpretivist)	Positivist	Critical, interpretivist
Ways of knowing	One	Many
Aim and design		
Aims at improvement	Yes	Yes
Aims to measure effectiveness of a clinical intervention	Yes	Yes, among other things
Co-design of research plan, involving participants	No	Yes
Participatory and democratic	No	Yes
Controls for bias and confounding factors	Yes	No
Accounts for and investigates context, social processes, patient engagement, equity	No	Yes
Measures context-dependent interventions and interactions	No	Yes
Incorporates complexity	Limited	Yes
Creates communicative space	At design phase	Throughout
Methods		
Quantitative methods	Yes	Not necessarily
Qualitative methods	No	Primarily
Blinding	Yes	No
Intervention improvement via cyclical iterations	No	Yes
Results/findings		
Design adjusted concurrent to emerging findings	No	Yes
Derives data and results from practice of reflexivity	No	Yes
Emergence (new, unexpected/expected knowledge)	Yes, as incidental findings, unintended consequences	Yes, as emergent findings specifically sought
Results in immediate multidimensional change	No	Yes
Results in later change in clinical practice	Yes	Yes

The purpose of this article is to examine the use of AR cycles and characteristics as a frame for designing and executing a telemonitoring RCT. The case we use in our argument aimed to assess the effect of telemonitoring on health-related quality of life, self-care, hospital use, costs and the experiences of patients, informal carers, and health care professionals. We present background literature, a telemonitoring RCT as the case study, and a critical analysis of our AR experience related to the case study.

### Acquiring evidence for practice

RCTs are not enough for acquiring evidence and applying it in practice. It can take up to 17 years for research from RCTs to become everyday clinical practice, because of repetitive research projects, and difficulties in identifying relevant research for practice [7]. Research into telehealth is not compatible with that timetable, as technology is constantly changing as refinements and innovations emerge [7, 8]. Kaldoudi, Chatzopoulou, and Vargemezis [9] argue that RCTs alone may not be enough for telehealth because the intervention is more than the technology. Furthermore, the intervention can influence and be influenced by uncontrollable confounding factors, e.g., local context of the patient, the organisation, and healthcare processes. Bias cannot be controlled because telehealth RCTs cannot be blinded - it is obvious to participants that groups with and without the technology are being compared [10].

Telehealth consists of complex interventions. The UK Medical Research Council’s (MRC) evaluation framework defines complex interventions as having a number of intervention components, and associated behaviours, groups, organisational levels, intervention flexibility, and number and variability of outcomes [11]. Telehealth research is usually complex, involving patients doing self-care, people being cared for at a distance with complex processes that are not bounded by the tyranny of geography or time, and extend beyond the patient-clinician relationship and a single organisation, to include multiple members of the multi-disciplinary team and the technology vendor.

Complex interventions require complex evaluations. Different methods can and should be used to evaluate interventions, and RCTs have a place, but do not sufficiently evaluate complex interventions, especially those that involve ICT. Lewin, Glenton and Oxman [12] conducted a systematic literature review of RCTs that included qualitative research, revealing that there is usually no overt link between the qualitative report and the RCT. The qualitative data is usually reported in the form of feasibility studies prior to an RCT. Campbell, Fitzpatrick, Haines, et al. [13] present a design for complex RCTs that includes a staged approach and collecting qualitative data, but does not address the difficulties associated with lack of blinding, and the rapid development of technologies. Mohr, Cheung, Schueller, et al. [7] propose a rapid cyclic form of RCT, but their method is limited to rapidly developing mobile technologies with high levels of sign-up and attrition. Murray, Teweek, Pope, et al. [14] propose that normalisation (i.e. an intervention becomes a normal part of everyday work) should be the goal of intervention evaluations such as RCTs. Their Normalisation Process Theory provides a framework for intervention designers to focus on implementation, such as context and change impact before a trial begins. Although they advocate for co-design, implementation considerations, and reflexivity (which are also AR features), once the trial begins these activities are set aside until the trial’s results are available. The AR feature of returning the results to the participants as part of the research is lacking.

Greenhalgh, Russel, Ashcroft, et al. [4] argue that ehealth evaluations require more than experimental cause and effect measurement because of the richness of what can be learned from the particular in complex situations. The MRC evaluation framework [11] has potential for thick description that can arise from AR. The MRC framework is a cycle consisting of feasibility study, evaluation of effectiveness and change process, implementation (implementation, surveillance and follow up), and development (theory, modelling and evidence base development). There are opportunities for gathering the rich data that falls around RCTs, but without the properties and processes of Action Research, these opportunities to apply learning from each step of the cycle and effect change can be lost.

Since telehealth interventions are complex, and although RCTs are increasingly built to accommodate complexity, the evaluation of these interventions requires a fuller, richer and therefore thick description of the intervention and its effects. The acquisition and use of evidence should be part of the research process, built in from the first design step, and fed back to practitioners at every stage of the research. We propose Action Research as a framework to address the limitations of RCTs in evaluating telehealth interventions.

### Action research and the RCT

Action Research (AR) is an approach that can be used to frame research methods, adding dimensions of learning, developing and testing theory, and applying that learning in subsequent cycles [15]. AR is defined as

*‘a participatory, democratic process concerned with developing practical knowledge in the pursuit of worthwhile human purposes….It seems to bring together action and reflection, theory and practice, in participation with others, in the pursuit of practical solutions to issues of pressing concern to people, and more generally the flourishing of individual persons and their communities.’* [16]

AR is usually described as a cyclic research process in which participants plan, act, reflect on the results of their actions, and modify the plan for the next cycle, repeated until they achieve their goal [17]. Research and action are conducted together, the research informing and informed by each cycle of action. All this is done in a mutually agreed upon ethical framework [18]. AR involves participants in defining a problem and designing and testing potential solutions, e.g. interventions, service improvements, trial methods. This is more than co-design and could provide useful input to build a strong RCT design that results in successful intervention implementation and return of research results to the involved community.

Reason [5], describes five characteristics of AR:

1. Create a communicative space at the beginning of a project. Participants are identified and agree to contribute to the project’s direction and content.
2. Iterative improvement is core to an AR project. It sets the tone for the change desired by the participants.
3. Emergence occurs as a result of informed action – as research produces knowledge and shared understandings, new (expected and unexpected) knowledge can be incorporated into subsequent iterations.
4. There are different ways of knowing, many perspectives. Each way of knowing is valid.
5. Democracy and participation are the foundation of AR.

## Methods

### The case: telemonitoring for long term condition management

We designed an RCT aimed at assessing the effects of telemonitoring on health-related quality of life, self-care, hospital use, costs and the experiences of patients, informal carers and health care professionals [19]. The RCT intervention consisted of telemonitoring of vital signs measured by participating patients plus patient answers to daily questions about symptoms. Data were collected in an information system, which the research nurses assessed and responded to according to an established clinical protocol as described by Kenealy et al. [19].

Participating patients in the intervention arm were given the equipment and trained on how to use it. The control patients continued to receive ‘usual care’ by their clinicians. There were three sites. Site A was urban and the patients had heart failure. Site B was also urban and the patients had Chronic Obstructive Airways Disease (COPD). Site A and B patients were recruited from hospital specialists. Site C was rural, and the patients were recruited from general practitioners, regardless of diagnosis. These patients were mostly diabetic.

The RCT participants fell into two groups as depicted in Table 2.

**Table 2:** RCT participants grouped as healthcare providers and consumers

Participants	Site A (heart failure)	Site B (COPD)	Site C (diabetes)
Healthcare providers	1 cardiologist	3 respiratory physicians	2 general practitioners
	3 heart failure nurse specialists	2 respiratory nurse specialists	1 practice nurse
	1 hospital manager	1 respiratory nurse specialist with prescribing rights	1 rural health nurse
		1 hospital manager	1 kaiawhina (community health worker)
			2 managers
Healthcare consumers	49 telemonitoring hospital consumers with congestive heart failure	24 telemonitoring hospital consumers with chronic obstructive pulmonary disease	25 telemonitoring primary care consumers with diabetes and multiple long term conditions
	49 usual care hospital consumers with congestive heart failure	24 usual care hospital consumers with chronic obstructive pulmonary disease	0 usual care consumers

During the planning phase of the RCT a small sub-group of the researchers negotiated the use of AR to frame this complex RCT. The goal was to use stages and sub-stages of the RCT plus the AR characteristics to enhance the implementation of the intervention and return the results of the study as soon as possible into practice. While it was considered good practice by the research team to negotiate the intervention design, the AR advocates were not able to negotiate the inclusion of potential RCT participants (i.e. specifically the patients/consumers) to co-define the research problem and participate in the co-design of the intervention. At the time it appeared appropriate to exclude potential RCT participants (consumers) because of the requirement to eliminate confounding factors and bias – inclusion was considered to undermine the RCT purpose, design and results. We did not manage to negotiate some of the funding to be focussed on the AR aspects of the fully funded RCT process and activities.

The academic who initiated the RCT project recruited clinicians, managers and community leaders from his extensive network to participate as co-researchers in the initial stages. There was a collaborator from each site – two were specialist clinicians and the third site was represented by a manager. Once the design had been established for the purpose of funding applications, these people became participants in the different sites that they belonged to, and are represented as such in Table 2. These three people remained in the AR team as well.

The research team was large and multidisciplinary. There were 13 academics from two faculties (Medical and Health Sciences, and Business), and covered the fields of medicine, nursing, health informatics, general practice, population health, and accounting and economics. A separate project team was established to support the funding applications, procurement of the telemonitoring equipment and information management system, and provide budget and project management services. For the purpose of this article, the AR team consisted of the academics, project manager, and site collaborators, totalling 17. The vendor supplied and supported the technology but did not become a member of the AR team as they were contracted to provide a service. The research was described and funded as an RCT, not an AR project, although we negotiated AR as a frame.

The research group became the AR group, and persisted for the duration of the research project, i.e. October 2008 until June 2012, as per Table 3. The RCT itself ran from September 2010 until August 2011.

**Table 3:** Research timeline and activities

Timeline	Oct 2008	Early 2009	Nov 2009	Sept 2010 – Aug 2011	Ended Jun 2012
Main activity	Identify and define the research problem	Ethics approval	Procure technology	Execute RCT	Project close-off
				Site A: Sep 2010 – Aug 2011	
				Site B: Dec 2010 – Apr 2011	
				Site C: Sep 2010 – Feb 2011	
Objectives	Design research protocol	Refine protocol	Funding granted	Refine and finalise RCT protocol	Analyse data
	Recruit researchers	Identify participants	Submit tender	Enrol patient and clinician participants	Retrieve monitoring equipment.
	Apply for funding	Confirm research team	Recruit vendor	Recruit research assistants	Reports in journals
		Confirm data gathering tools	Refine RCT protocol	Use suite of data gathering tools	Disseminate results to participating District Health Boards
		Confirm recruitment processes	Refine clinical pathways	Analyse data	
			Confirm patient and clinician participant recruitment process		

In summary, three groups formed. The researcher group consisted of academics, some of whom were initially also clinicians from the different sites. Once the design was clarified, clinicians from Sites A and B shifted to the healthcare provider participant group, and the manager from Site C joined the researchers. The healthcare provider participant group was considered to be participants in that that they provided clinical services associated with the telemonitoring. They recruited patients, who made up the third group of consumer participants.

## Results

### AR as a frame

**Fig. 1:**
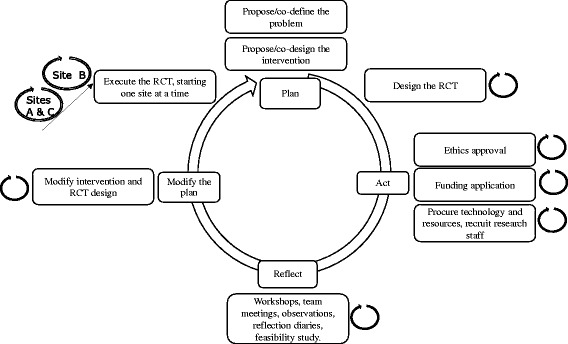
RCT framed by AR as a cyclic research process consisting of sub-cycles

Action researchers use a cyclic approach – plan, act, reflect and modify the plan for future cycles, and then repeat the cycle [17]. The repeating cycles are not limited to repeated content. Cycles change as new aspects emerge due to learning from previous cycles. Our AR team applied the principles of the cyclic learning process to inform each step of the planning and execution of the RCT, as depicted in Fig. 1. Reflection, learning and improvement were the goals, including the capacity to adopt what was learned into practice. The overall research project was treated as a single AR cycle, with sub-cycles. The problem definition and intervention design were done by the research team. Inclusion of the site clinicians and mangers in the research team in the early stages of the overall project was deemed appropriate for problem co-definition and intervention co-design.

**Fig. 2:**
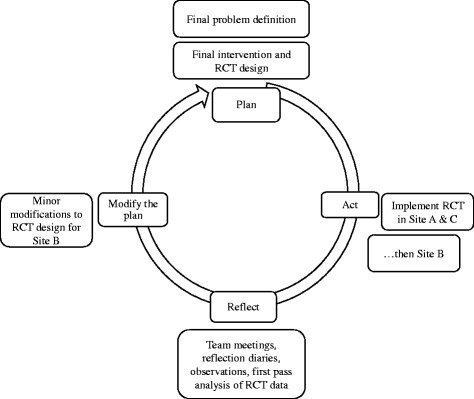
Site A, B, and C implementations as an AR cycle

Execution of the RCT then became a set of AR cycles as per Fig. 2. The implementation of each arm of the RCT at the three sites was each considered a sub-cycle, with learnings from the first sites (A and C) being implemented when the second site (B) was activated. Sites A and C were started in the same month. Learnings were exchanged between the sites until enrolments were standardised and initial problems had been resolved. Learnings from Sites A and C were then applied to site B when it joined the trial. The adjustments aimed to improve the RCT and intervention implementation, and did not result in material changes to the RCT design itself.

As the overall research project progressed, and sub-cycles of the intervention were implemented, the degrees of distance between the research team and the research nurses executing the intervention increased, resulting in lost opportunities for AR characteristics and cycles to be applied. Since the AR aspect of the project was not funded, the discipline of reflexivity, documenting observations and lessons learned, and the contributions of participants, were not built into the research design or followed up. As the project progressed the commitment to AR reduced as the commitment to the RCT increased.

Meetings commenced in the first cycle (starting in 2008) and continued until after the study finished in June 2012. Reflection diaries [20] were not included in our project, although they are standard practice in AR. During this time the emphasis progressively shifted from planning the RCT to interpreting data, planning for the dissemination of findings, and future implementation of telehealth in New Zealand.

### AR characteristics to co-design an RCT

Retrospectively we were able to see how the five characteristics of AR could be used in the design and execution of a telehealth RCT and the dissemination of the results.

1. Creating a communicative space to achieve co-design

Creating a communicative space within the research team occurred during the funding and ethics activities, and was sustained throughout the project in different ways, as per the objectives in Table 3. Funding and ethics approval applications required discussions and collaborative decision making and writing. Once funding had been approved a workshop was held to further develop relationships with a broad range of healthcare service leaders, academics and others who wanted to participate. This workshop attracted attendees from several sites all over New Zealand; not all attendees were able to commit their service to the project but wanted to participate in its planning.

Once the communicative space had been created the research and healthcare provider groups finalised which services would participate and under what circumstances, e.g., one urban site (Site A) wanted to include patients with heart failure, while a clinician from another urban site offered his COPD service (Site B). In contrast, the participants from the rural site were from a remote community, Māori, and had diabetes (Site C). Terms of engagement for Site C followed a collaborative and participative protocol that involved community elders, and a review of the research protocol prior to formal engagement in the project. This process leveraged the democracy and participation characteristic of AR by means of Kaupapa Māori [21]. Smith argues that “Kaupapa Māori is a 'local' theoretical positioning which is the modality through which the emancipatory goal of critical theory, in a specific historical, political and social context, is practised” [22]. Kaupapa Māori research reflects a Māori world view privileging Māori values in the interests of developing a research framework that is culturally safe. Kaupapa Maori research can strengthen community relationships, particularly when the research is driven by those who live in the community of interest. As Maori seek to be self-determining, Smith argues that such research allows for solutions and interventions that can change and improve lived realities.

The first meeting with aspiring research leaders and participants (the workshop) established the communicative space, although it was not a named AR workshop. Once the funding and ethics approval had been obtained, weekly and then fortnightly meetings among the researchers occurred. These meetings were a time for updating one another (communicative space), reflection on progress and problems (step 3 of the AR cycle), problem solving, decisions about adapting the research design and/or providing feedback to participants from the reflections, and determining next steps for the research to progress. During this time clinical processes were documented and modified for the purpose of implementing the telemonitoring intervention. These regular meetings of the broader research team discontinued once the RCT participants began being enrolled, and focussed irregular meetings occurred with a smaller team that was primarily focussed on the trial itself.

2. Different ways of knowing, many perspectives

The research group was a large team with 17 members who came from different research perspectives (positivist, interpretivist, critical). At times conflict arose from perceptions of incommensurability of these different perspectives, as well as interpersonal conflict. There was heated debate about contextual data collection and the value (or lack thereof) of qualitative data collection, e.g. patient perceptions of the technology. Stressors, such as research assistant turnover, the complex and complicated nature of the research, and differences in opinion, resulted in periodic interpersonal conflict, which was mostly resolved when participants were redirected to the common cause of their endeavours, i.e. the purpose of the research project.

3. Democracy and participation

Democracy and participation refers only to the research/AR group as described above. The researchers had different interests in the RCT and were asking different questions, e.g. consumer perspectives, technological aspects, costs and economics, management, clinical outcomes. Each researcher’s contribution was valued.

The leaders and managers in the remote site (Site C) took AR a step further, insisting on participative decision-making throughout the study, extending it to participating clinicians and patients using the principles of Kaupapa Māori Research [21]. As reported in [19] this rural community was “…an hour’s drive away from a regional city, in an impoverished area with a high proportion of indigenous Māori. Patients nearly all had type 2 diabetes and most had other long-term conditions. [Site C] contributed only intervention patients [i.e. no control patients], as the area is underserved and they wanted to use the opportunity to increase services for their patients and considered it unethical to enrol their patients into a control group.” Contrary to predictions that patient participation would undermine the RCT results, the differences between the sites and the lack of a control arm in the remote site did not appear to influence the trial results [19], but the outcomes of the opportunity to leverage AR practices and characteristics were limited to the site itself.

4. Constant iterative improvement

Constant iterative improvement was achieved by using AR cycles (Figs. 1 and 2). The project manager used the typical project management PDSA cycle (plan, do, study, act) [23], to ensure that this complex RCT was appropriately executed.

We were obliged to use the New Zealand government’s procurement process to secure a vendor for the telemonitoring equipment. This process was drawn out and complex, and one of the incidental outcomes of the research process was a contribution to the development of a simpler process, ‘active procurement’ [24]. The development of ‘active procurement’ could be attributed to the AR reflexivity that was being practiced in the early stage of the project. Such incidental outcomes (emergence) are common with AR [5].

We learned from each cycle and sub-cycle of the entire project. Refinement of clinical guidelines, improvement in the functioning of the technology, improvements as we progressed from one site implementation to the next, all produced learning that was used to inform the next stage of the project. Refinement of the RCT protocol and associated processes in Fig. 2 occurred in sub-cycles as each of the two urban sites began and completed their data gathering stages. Once a group of participants was locked into the RCT intervention and data gathering there was limited opportunity to use the AR cycle because these had to be consistent among participants. We used the AR cycle of learning and application but were only really successful in the first half of the research project.

5. Emergence

Emergence is to be expected in AR. The cyclical constant improvement approach enabled us to look for consequences and address any adverse events early. One should expect unintended consequences to emerge in RCTs, and more so in health information technology research [25]. We learned that the technology, despite being among the best available internationally at the time of selection, failed in a number of ways, e.g. lost data. Data quality can be compromised when patients are required to enter data themselves, rather than allow the system to automatically collect and transfer vital measurements. New processes of care emerged for remote patients (Site C) who otherwise would not have had access to service, but problems associated with distance and the need for additional support were also identified. An RCT focussed solely on the intervention may not have given sufficient space to identify these issues and address them and learn from action taken. Sites A and B started their version of the RCT at a different time from Site C (Table 3), taking advantage of what was learned from unintended consequences and continuous improvement emerging in previous months.

### Successes and challenges of using AR to frame an RCT

Change is the main reason for AR. The desire for change arises from a critique of the current situation and the view that there could be something better in its place. This concurs with the motivation for RCT-based research – to identify and examine an intervention that has the potential to improve the health of individuals and/or communities. In answering the case’s research question our aim was to influence national policy on the development of telehealth as a valid, funded, and cost-effective choice for delivering health services that have a likelihood of improving health outcomes for people with chronic conditions. As the patients and their clinicians and service managers learned from their participation, processes and attitudes changed, and improvements occurred, thereby translating research into action. Many of the patients who participated were reluctant to return the telemonitoring equipment as they claimed it had made a difference in their lives. This raised an ethical debate about the consequences of withdrawing a research intervention when a project (and its funding) comes to an end.

We were new to applying AR characteristics and cyclic iterative learning to quantitative studies. At times this pluralism was natural and at other times the interpretivist nature of AR and positivist underpinning of RCTs seemed incommensurate, e.g. potential patient participants were excluded from co-definition of the problem and co-design of the intervention and RCT. For the most part the different approaches worked well together and offered opportunities for more rigour, stronger analysis of the data, and more useful findings. We did experience some challenges. Some of the researchers were strongly positivist, making it difficult for them to see value in what they saw as subjective, biased, and unreliable practices, e.g. positioning researchers inside the research and applying results as they become available. The positivist requirement to generalise findings that are bias free appears to be incommensurable with the interpretivist requirement for transferability and trustworthiness of findings. The main challenge was the lack of funding to build AR deliberately into the overall research project, ensuring that the discipline of practicing and documenting reflexivity was regularly practiced in meetings. The effects of adjusting the intervention during implementation (after learning from the implementation at other sites), and the effects of the research on the organisational context, could have been more rigorously investigated and reported. This could have informed future decisions about including possible patient participants in the early stages of the research. A significant challenge was the increasing degree of distance (and decreasing degree of influence) between the researchers and implementers of the trial, limiting the opportunities to document, analyse, share and apply any influence of AR practices on the trial.

The project leadership changed, resources became scarce for continued participation of academics, and the research/AR team shrank as researchers and collaborators turned to the demands of their daily work lives because the discipline of AR was not documented in the research project’s plan. The opportunities for AR contributions from those who initially negotiated AR as a frame became limited and as funding became more focussed on the RCT cycle, the Action Researchers withdrew.

## Discussion

An RCT was conducted to assess the effect of telemonitoring on health-related quality of life, self-care, hospital use, costs and the experiences of patients, informal carers and health care professionals. AR was negotiated as a frame for the RCT and was conducted by Action Researchers new to using AR to frame an RCT, with mixed levels of commitment from the research project’s team members.

We were able to apply an AR cycle to the process of developing and executing an RCT design, and implement sub-cycles to each step of the overarching cycle (Figs. 1 and 2). Initially we aimed to only use the AR cycle but found ourselves using AR characteristics as well. The characteristics of AR were applied, especially in the early stages of the project. AR offers ways to deliberately and systematically collect data that is not usually collected in RCTs. It is interesting to note in our RCT report that although the RCT itself did not reveal a strong argument to implement tele-monitoring, the qualitative data that was collected revealed that tele-monitoring is a valuable intervention, especially from the patient’s point of view [19].

Why bother with AR? AR and RCTs complement one another in many ways, as can be seen in Table 1. We know that RCT is a good way of measuring the effect of a telehealth intervention, and we know how important it is to design an RCT well [26]. In the critical approach the researchers are saying that the situation under study should be better in some way, i.e. they critique the status quo, identify a vision for improvement and, using AR cycles and characteristics, improve the situation [27]. This could include an RCT to examine the effectiveness of their actions as a sub-cycle of an overall AR cycle [28]. On the other hand, there is a need to extend the scope of an RCT to include other data about the phenomenon being studied, e.g. to deliberately include the data that falls around an RCT that is often discarded due to concerns about bias and confounding factors [29]. In the interests of quality, the process of defining the scope and purpose of an RCT should be documented for other researchers to use: AR offers a rigorous approach to this documentation and process.

AR appears to be at odds with positivist research [28, 29], and this was a challenge at times during our project. Critical, interpretivist and positivist ways of knowing could be seen as mutually exclusive [28, 30]. They could complement one another, as natural responses to different types of knowing [8, 31]. Positivist research is good for identifying efficacy of clinical interventions where causal links are useful in evidence based care. A broad range of theories about telehealth (including tele-monitoring) are therefore embraced, e.g. socio-technical (e.g. technology acceptance model by Davis), health behaviour, and economics theories [32]. AR thus embraces different ways of knowing to enable multiple aspects of an intervention to be researched simultaneously, while also informing the actual implementation of the intervention in the context of an organisation [33]. Including patient participants in the co-definition and co-design of an RCT was initially seen to compromise the execution of the RCT. Instead of conducting a feasibility study, one could include those participants in the early stage of an RCT design process to strengthen the focus and applicability of the intervention and the RCT itself.

The inclusion of evidence from RCTs into evidence based care is challenging. Clinicians are unable to absorb the rising number of RCT publications, of which the quality is variable [34]. Furthermore, there is a time lag between the performance of an RCT, its publication and the use of its results in everyday clinical practice, further complicated by the slow moving organisational and regulatory environment in which healthcare is practiced [29, 34]. AR offers opportunities to initiate change where change can be implemented as the result of incremental, cyclic improvements. Formative evaluation that is concurrent to an RCT offers opportunities for change in medical practice that are not available in the summative orientation of the RCT process itself [29].

Formative and summative evaluation of a tele-monitoring intervention, framed by AR, and involving Information and Communications Technology (ICT), implies a form of ‘action design research’ [35]. This approach aims to meet the needs of practitioners by designing an ICT artefact for organisational implementation, while simultaneously conducting theoretical research using formative and summative research methods. ICT is not an intervention in the same way as a medication or surgical procedure is; it is an ‘ensemble artefact’ incorporating clinical and organisational processes and structures into the technology [35]. Consequently, when measuring the tele-monitoring impact on clinical outcomes for people with long term conditions the research data generated represents the embedded processes and structures as well as cause and effect links.

An RCT is not enough to demonstrate whether the ‘intervention’ works or not [36]. It is not enough to design complex RCTs as proposed by Campbell et al. [13], or to create continuous RCTs to accommodate rapid technological changes as proposed by Mohr et al. [7]. It is not enough to conduct a feasibility study or to simply include qualitative analyses arising from an RCT [12]. We propose that the AR process and characteristics enable telehealth researchers to evaluate formatively and summatively, and enable research teams and collaborators to bring about change as and when opportunities arise without compromising the quality of an RCT.

AR as a frame for planning and executing RCTs offers a structure, processes and characteristics. Researchers with different takes on the research question are able to explore their different ways of knowing, e.g. organisational researchers using social sciences, technology researchers using socio-technical theory and clinicians using positivism to establish causal effects. Action research and positivism both aim to improve a situation. At times AR and RCTs are at odds with one another, but with AR as the overarching frame that allows for democratic planning (and permits different types of participants), responsiveness to emergence at appropriate times, and iterative learning and emergence, then the data that falls around an RCT can, and should, be used purposively.

## Conclusion

Our research team agreed to use AR to frame an RCT to assess the effect of tele-monitoring on health-related quality of life, self-care, hospital use, costs and the experiences of patients, informal carers and health care professionals. We were new to the idea of deliberately framing an RCT as an AR project and we learnt how to work together within a communicative space to build an RCT that reflected different ways of knowing. It was more than a mixed methods study. Our study accommodated theoretical pluralism (although challenging at times, especially when the different approaches appeared to be philosophically incommensurable, and when funding limited our capacity to use the discipline associated with AR) to enable researchers representing different ways of knowing. At times doing an RCT framed by AR seemed clumsy, but the way the cycles in Figs. 1 and 2 worked meant that certain ways of knowing dominated depending on the purpose of that cycle.

We propose that the cyclic framework plus the characteristics of AR should be used in the planning, execution and close-off of RCTs to enable stronger design, richer data collection and earlier adoption of evidence into practice for patients, clinicians, the multi-disciplinary team, investors, and improvement of the healthcare environment [37]. AR and RCTs share a purpose – to make improvements. AR enables clinicians and other healthcare practitioners to contribute to research and return the results into everyday practice. Based on our experience, we recommend that all RCTs could be framed as an AR project to enhance the quality, value, and impact of the research on everyday health care.

## Abbreviations

AR, action research; COPD, chronic obstructive pulmonary disease; ICT, information and communication technologies; PDSA, Plan, Do, Study, Act (cycle); RCT, randomised controlled trials
